# Pathology of Chorea

**Published:** 1890-02-15

**Authors:** 


PATHOLOGY OF CHOREA.

Dr. Archibald E. Garrod writes to the *Lancet*, November 23rd, on the pathology of chorea, and remarks that there is good reason for believing that chorea is in many cases a manifestation of rheumatism. The lesion most intimately associated with eudocarditis is the formation of subcutaneous nodules. Like this nodule formation, chorea is intimately associated with eudocarditis. The class of patients most subject to chorea is precisely that in which there is most tendency to nodule formation. There is some basis for the hypothesis that chorea is the outward sign of a lesion, and has its origin in a temporary overgrowth of connective tissue in the nerve centres, of the same nature as the subcutaneous nodules of rheumatic origin.

